# Quantitative analysis of brass compensators for commissioning of the Pinnacle planning system for IMRT

**DOI:** 10.1120/jacmp.v16i6.5531

**Published:** 2015-11-08

**Authors:** Larry L. Gates, David J. Gladstone

**Affiliations:** ^1^ Norris Cotton Cancer Center Geisel School of Medicine at Dartmouth Lebanon NH USA

**Keywords:** brass, compensators, IMRT, Pinnacle, commissioning, gamma, dose, distribution

## Abstract

Brass compensators for beam modulation present an alternative method for IMRT treatment planning to traditional dynamic leaf modulation. In this work, we present a detailed method to commission the Pinnacle treatment planning system for IMRT using brass compensators. Beam attenuation from various brass thicknesses were measured using an ion chamber, as well as a MapCHECK device, for a representative seven‐field IMRT plan. We show that Pinnacle's parameters for compensators can be optimized to match the measured results while still maintaining the original open‐beam model. Only Pinnacle's modified scatter factor and brass density value were adjusted. Beam attenuation in several brass slabs were optimized by minimizing a $\chi^{2}$ function of measured and modeled data for several depths in solid water. Using MapCHECK, Pinnacle's parameters were optimized by minimizing the number of points that fail during a gamma analysis of seven fields in a typical IMRT plan. Our results show that Pinnacle's 10X beam is best modeled using a brass density of $7.8\;g/{\text{cm}}^{3}$ and a modified scatter factor of 0.1 ${\text{cm}}^{-1}$.

PACS numbers: 87.56.N, 87.56.ng, 87.55.kh, 87.55.de

## I. INTRODUCTION

Today's modern regimens of intensity‐modulated radiation therapy (IMRT) are typically achieved with MLC based compensation. Highly conformal dose constraints and dose escalation can be achieved through beam compensation by solid attenuators (i.e., brass) or by MLC. In the latter, multiple moving tungsten alloy leaves create a summed attenuation of the beam as a function of field dimensions, x and y, that results in a modified beam intensity reaching its target. Using solid attenuators, a compensator is fabricated by milling the material such that the thickness of the material varies over the field dimensions, resulting in a modified beam fluence, as well.

Inverse planning algorithms are used to compute the necessary attenuation required as a function of gantry and collimator angle, given the 3D dose optimization parameters by the user. Once the ideal fluence is computed, a leaf sequencing algorithm attempts to achieve this ideal fluence by specifying a series of MLC patterns. In the case of brass, an algorithm specifying a 3D compensator shape is used.

We consider here the use of solid brass compensators for IMRT plans using the Pinnacle (Philips Radiation Oncology Systems, Fitchburg, WI) treatment planning system (TPS). Various methods have been used to commission TPS for brass; however, there are very few publications that utilize several brass thicknesses for modeling, and some change the open field characteristics of the TPS beam model. In this work, we keep the open field modeling constant while changing only the Pinnacle parameters affecting the brass compensator. These changes are based on a $\chi^{2}$ optimization from three brass slab thicknesses, as well as a separate MapCHECK (model 1175, Sun Nuclear Inc., Melbourne, FL) optimization from brass compensators from a typical IMRT plan.

The Pinnacle TPS is model‐based,^(1)^ using several parameters that are adjusted by the user to improve agreement between its calculation model and a set of measured profiles. The best fit model is used to compute dose distributions using a superposition–convolution kernel. Brass, or solid compensators, can be modeled in Pinnacle using two adjustable parameters: density $\left(g/{\text{cm}}^{3}\right)$, ρ, and a modified scatter factor, MSF, which is a correction factor ranging from $0-1.0\;{\text{cm}}^{-1}$ which accounts for extra scatter from the compensator material as compared to tissue.

It is possible to adjust the open‐beam parameters, as well, to account for compensator effects; however, such a change would compromise the integrity of the open‐beam model. We feel that isolating a model in Pinnacle just for brass compensator planning creates a significant risk that it might be used in error for non‐IMRT planning. Further, we should expect that any brass model would collapse to provide the open‐beam characteristics when the IMRT algorithm calls for zero brass thicknesses for a particular position (x, y) within the compensator.

In this work, we analyze the effect of the MSF and density value on Pinnacle's dose attenuation prediction through slabs of brass, as well as Pinnacle's dose distribution using a MapCHECK device.

There are a few publications dealing with TPS optimization for brass compensators. Oguchi and Obata^(2)^ have commissioned the XiO TPS for 4 MV and used an in‐house milling machine for brass compensator fabrication.

Cubic tungsten blocks $0.5\times 0.5\times 0.5\;{\text{cm}}^{3}$ have been assembled to form a larger ‘block‐piled’ modulator and is described in a paper by Sasaki and Obata.^(3)^ Their work looked at 4 MV and 10 MV on a Pinnacle TPS.

In a paper by Opp et al.,^(4)^ commissioning for Pinnacle is described for brass compensators by identifying a single "most probable" brass filter thickness and then using this for beam modeling. A separate 6 MV beam model for brass was thus obtained. By contrast, our work presented here maintains the original open field model and uses several brass thicknesses to optimize the parameters. Our density and MSF are similar to the results obtained by Opp et al.^(4)^

## II. MATERIALS AND METHODS

### A. Optimization using attenuation through brass slabs

Three brass slabs of thicknesses 1 cm, 3 cm, and 5 cm were manufactured by .decimal, Inc. (Sanford, FL). We measured the attenuation of a 10 MV beam through the slabs using a Varian 2100EX linear accelerator (linac) (Varian Medical Systems, Palo Alto, CA). The slabs were mounted on acrylic trays which fit into the linac accessory mount.

The beam was measured using an Exradin A12 ion chamber (Standard Imaging Inc., Middleton, WI) at several depths (dmax=2.5 cm, 10 cm, and 20 cm) in water using a Wellhofer Blue water tank (IBA Dosimetry, Schwarzenbruck, Germany). Several field sizes ($5\times 5\;{\text{cm}}^{2}$, $10\times 10\;{\text{cm}}^{2}$, and $20\times 20\;{\text{cm}}^{2}$) were measured.

Attenuation of the beam as a function of brass thickness should follow the standard attenuation equation:

(1) $$I(x,y)=I_{O}(x,y)e^{e^{-u\times t(x,y)}}$$

where *I* is the attenuation of the beam, $I_{O}$ is the open field reading, *u* is the effective attenuation coefficient, and *t* is the brass thickness as a function of field position *x* and *y*. A determination of u must consider beam hardening and beam divergence and is well described by Chang et al.^(5)^ Here, we consider only the attenuation at central axis and scatter effects, such that the logarithm of the ratio $I(x=0,y=0)/I_{O}(x=0,y=0)$ should yield a straight line when plotted as a function of brass thickness. There will be some deviation from linearity due to beam hardening and scatter; however, we are not attempting to determine u, but rather compare the measured results with that predicted by the Pinnacle planning system, and optimize the latter. A simple calculation of the $\chi^{2}$ value, or "goodness of fit", of our measured values and our predicted values, will guide us in determining the optimum parameters to use in Pinnacle.

Using the logarithm of I(brass thickness)/I(open field), we compute $\chi^{2}$ values using

(2) $$\chi^{2}=\frac{{\left(p_{1}-x_{1}\right)}^{2}}{x_{1}}+\frac{{\left(p_{2}-x_{2}\right)}^{2}}{x_{2}}+\ldots$$

where $x_{i}$ are the measured values and $p_{i}$ are values obtained from Pinnacle for i=1,2,3 representing brass thicknesses 1 cm, 3 cm, and 5 cm, respectively. The optimum parameter to use for brass compensator IMRT will be the minimum $\chi^{2}$ for the dataset generated as a function of Pinnacle parameters MSF and ρ.

### B. Optimization using gamma analysis on a typical seven‐field IMRT plan

A typical seven‐field IMRT plan was generated using Pinnacle on a standard IMRT Head and Neck Phantom (CIRS, Norfolk, VA). This step was performed as a comparison to the brass slab optimization to confirm accurate modeling with real compensators with large peaks and valleys where complex photon scatter may be present with respect to slab tests.

Peaks and valleys in a typical IMRT compensator will create sharp fluence gradients and scatter the photons more than a flat surface. We are, therefore, interested in optimizing the model using the results from a MapCHECK device. Using this device, we measured the output from each of the seven brass compensators from our IMRT plan. The compensators were fabricated by .decimal, Inc. The measured dose distribution was compared to the distribution predicted by our Pinnacle model by using the MapCHECK software's gamma analysis algorithm. The number of failing points, or diodes whose values failed the gamma test, was used to assess how well the model fit the measured results. A diode fails if its gamma value is >1.0. A threshold of 10% was chosen to exclude diodes outside of the IMRT field. MapCHECK computed the percent dose difference using the local value of dose, without choosing the "Van Dyk" setting.^(6)^ Similar to the brass slabs, only Pinnacle's model parameters MSF and ρ were adjusted.

In order to use the gamma analysis as a metric for modeling Pinnacle, we decided to look at the results for various, more stringent, values of DTA (distance to agreement) and percentage dose difference. A typical institutional criteria for gamma analysis^7^, ^8^ is to use values of DTA=± 3 mm and % absolute dose difference =±3%. These values give very good passing rates (as expected), but also show little change in the number of failed points as the parameters MSF and ρ are varied. Therefore, we decided to look at gamma values using several more stringent parameters of %dose‐difference/DTA of 2%/2 mm, 1.5%/1.5 mm, and 1%/1 mm. Using more difficult pass criteria should illuminate the differences among the adjustable parameters MSF and ρ.

## III. RESULTS

### A. Results of optimization using attenuation through brass slabs

**Figure 1:**
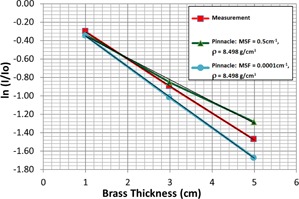
10 MV X‐ray attenuation through solid brass. Ion chamber readings in water at a depth of 10 cm, $10\times 10\;{\text{cm}}^{2}$ field size, are shown normalized to the open field reading and plotted as a logarithm to demonstrate the closeness to linearity as predicted by Eq. (1). Simulated data from the Pinnacle planning system is also shown for several scatter factors, MSF, to show the effect of changing this parameter. A linear least squares fit is shown as a thin black line, which superimposes well on the measured plot and the plot with $\text{MSF}=0.0001\;{\text{cm}}^{-1}$, indicating good linearity from these data.

Our measured attenuation through three brass slabs of thickness 1, 3, and 5 cm are shown in Fig. 1. Measured values from our ion chamber in water were taken with and without brass (open field) and the logarithm of the ratio was plotted versus brass thickness. These values are shown alongside the predictions from the Pinnacle TPS for two different values of the MSF.

The measured results show good linearity, indicating that beam hardening and scatter have only a small effect on these thicknesses. The small influence of beam hardening through compensators has been described by others.^(9)^ The Pinnacle model for $\text{MSF}=0.0001\;{\text{cm}}^{-1}$ shows similar linearity to the measured data, but shows too great an attenuation for all thicknesses. The Pinnacle model using a higher $\text{MSF}=0.5\;{\text{cm}}^{-1}$ shows significant nonlinearity, indicating that extra scatter from a high MSF in the model does not reflect the true beam characteristics.

**Figure 2:**
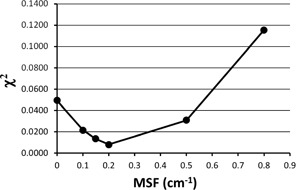
A $\chi^{2}$ calculation of Pinnacle's attenuation data and measured data is shown as a function of several values of the modulation scatter factor, MSF. The lower values of $\chi^{2}$ are a better "fit" to the measured data. An optimum value of $\text{MSF}=0.2\;{\text{cm}}^{-1}$ is seen from this graph when holding the density $\rho =8.498\;g/{\text{cm}}^{3}$ constant.

We will use the $\chi^{2}$ variable to optimize the Pinnacle MSF and ρ variables. For example, the $\chi^{2}$ value for the Pinnacle $\text{MSF}=0.0001\;{\text{cm}}^{-1}$ data in Fig. 1, is 0.0494, while for the $\text{MSF}=0.5\;{\text{cm}}^{-1}$ data, it is 0.0308, using Eq. (2). If we hold the density, $\rho =8.498\;g/{\text{cm}}^{3}$ constant, and plot the $\chi^{2}$ values for several MSF values, we obtain the interesting curve in Fig. 2. We find a minimum value for $\chi^{2}$ in Fig. 2 at $\text{MSF}=0.2\;{\text{cm}}^{-1}$, indicating the best fit to our measured data for a density of $\rho =8.498\;g/{\text{cm}}^{3}$.

**Figure 3:**
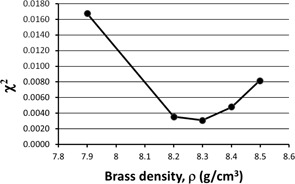
$\chi^{2}$ as a function of Pinnacle's model parameter for brass density. We see an optimum value of $8.3\;g/{\text{cm}}^{3}$ when the scatter factor MSF is held constant at a value of $0.2\;{\text{cm}}^{-1}$.

We can perform the same optimization by varying ρ and holding MSF constant. Figure 3 shows $\chi^{2}$ as a function of ρ while holding MSF constant at 0.2 cm^‐1^. From the $\chi^{2}$ minima, we obtain an optimum value of $\rho =8.3\;g/{\text{cm}}^{3}$ for $\text{MSF}=0.2\;{\text{cm}}^{-1}$.

**Figure 4:**
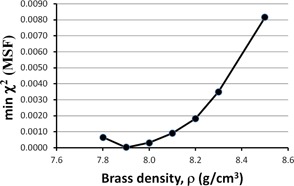
The minimum $\chi^{2}$ value plotted as a function of brass density. The minimum $\chi^{2}$ was obtained by computing the minima from several $\chi^{2}$ vs. MSF plots, as in Fig. 2, where MSF is the modulation scatter factor. The curve shows a minimum at $7.9\;g/{\text{cm}}^{3}$, which is the optimum parameter value in the Pinnacle model for flat brass slabs for a $10\times 10\;{\text{cm}}^{2}$ field size and a 10 cm depth in water.

We can continue to optimize in this fashion by recomputing $\chi^{2}$ versus MSF, holding constant the new optimized ρ = 8.3 g/cm^3^. However, we are interested in the minimum $\chi^{2}$ value, for each value of ρ. Plotting the minimum $\chi^{2}$ from $\chi^{2}$ versus MSF (holding ρ constant), as a function of ρ will reveal our final optimum ρ value to use. We present this in Fig. 4, showing data for a $10\times 10\;{\text{cm}}^{2}$ field at a depth of 10 cm. From this curve, we see that a density of $\rho =7.9\;g/{\text{cm}}^{3}$ yields a minimum $\chi^{2}$, indicating the optimum value for the Pinnacle model.

**Figure 5:**
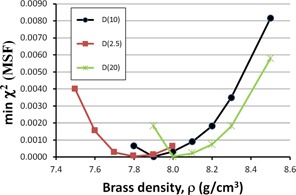
The minimum $\chi^{2}$ value plotted as a function of brass density. The data for a $10\times 10\;{\text{cm}}^{2}$ field size and several depths (2.5 cm, 10 cm, and 20 cm) in water were analyzed to demonstrate its effect on the optimum value for Pinnacle's model. The average minimum for the curves shown is at $7.9\;g/{\text{cm}}^{3}$.

**Figure 6:**
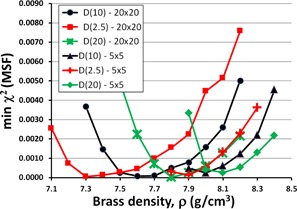
The minimum $\chi^{2}$ value plotted as a function of brass density for field sizes $5\times 5\;{\text{cm}}^{2}$ and $20\times 20\;{\text{cm}}^{2}$, and several depths (2.5 cm, 10 cm, and 20 cm) in water. Similar to those in Fig. 5, these data show the effect of field size and depth in water on the optimum parameter values for Pinnacle. The minima for the curves vary from $7.3–8.1\;g/{\text{cm}}^{3}$, with the average at $7.8\;g/{\text{cm}}^{3}$.

For depths and field sizes other than 10 cm and $10\times 10\;{\text{cm}}^{2}$, the values obtained for optimized ρ and MSF vary slightly, as seen in Fig. 5 for depths of dmax=2.5 cm, 10 cm, and 20 cm, and in Fig. 6 for field sizes of $5\times 5\;{\text{cm}}^{2}$ and $20\times 20\;{\text{cm}}^{2}$. Here, we see lower optimized ρ values for a $20\times 20\;{\text{cm}}^{2}$ field size and larger values for a $5\times 5\;{\text{cm}}^{2}$ field size. The field sizes shown here are typical for most IMRT plans. Considering the typical 10 cm depth used in most IMRT plans, the average ρ value among the curves for D(10) is $\rho =7.87\;g/{\text{cm}}^{3}$. For all the curves, the average is $\rho =7.83\;g/{\text{cm}}^{3}$. These values are very close given the data spacing in our acquisition, and therefore $\rho =7.83\;g/{\text{cm}}^{3}$ is a reasonable value to use for Pinnacle's IMRT optimization.

### B. Results of optimization using gamma analysis with MapCHECK

In the previous section, the brass slab optimization led to our Pinnacle model using parameters MSF = 0.001 cm^‐1^ and $\rho =7.83\;g/{\text{cm}}^{3}$. The scatter factor MSF is essentially not needed in the case of flat brass slabs, however, we expect to see some scatter through brass compensators that have more structure in them.

**Figure 7:**
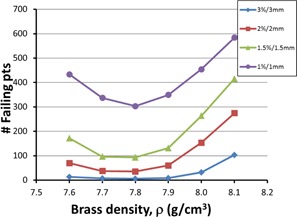
Optimization of Pinnacle's value for brass density using gamma analysis. Pinnacle's dose distribution for a typical seven field IMRT plan was compared with the measured MapCHECK results. The number of failing points represents the sum of the failing points for all seven fields. Four different levels of gamma analysis are shown, changing the % dose difference and DTA values. The more strict levels reveal the optimum value of brass density at $7.8\;g/{\text{cm}}^{3}$.

Optimization of the density parameter, ρ, was done by plotting the number of failing points versus ρ for four different gamma criteria. The MSF was held constant at $0.1\;{\text{cm}}^{-1}$. Figure 7 shows the results of the MapCHECK density optimization. Although the graph for gamma 3%/3 mm appears flat, the other curves show clearly that a minimum exists for $\rho =7.8\;g/{\text{cm}}^{3}$.

**Figure 8:**
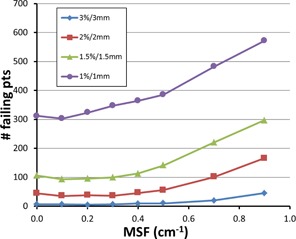
Optimization of Pinnacle's modulation scatter factor, MSF, using gamma analysis. The dose distribution for a typical seven‐field IMRT plan was compared with the measured MapCHECK results. The number of failing points represents the sum of the failing points for all seven fields. Four different levels of gamma analysis are shown, identical to Fig. 7. The more strict levels reveal the optimum value of MSF at $0.1\;{\text{cm}}^{-1}$. Density was held constant at $\rho =7.8\;g/{\text{cm}}^{3}$.

Using a constant $\rho =7.8\;g/{\text{cm}}^{3}$, we next optimize the MSF using the same gamma criteria. In Fig. 8, we plot the number of failing points versus MSF, and these show curves with a broader minima and closer to zero on the graph. They show a minimum at $\text{MSF}=0.1\;{\text{cm}}^{-1}$, with a small, but clear, increase in the number of failing points for $\text{MSF}=0.001\;{\text{cm}}^{-1}$. Values of MSF from $0.0–0.4\;{\text{cm}}^{-1}$ show similar results due to the broad minima, with the gamma 1%/1 mm curve having a more convincing minima at $\text{MSF}=0.1\;{\text{cm}}^{-1}$. Thus, we used values of $\text{MSF}=0.1\;{\text{cm}}^{-1}$ and $\rho =7.8\;g/{\text{cm}}^{3}$ as our final optimized parameters for the Pinnacle model for 10 MV.

## IV. DISCUSSION

The Pinnacle treatment planning system is a model based system that uses a 3D superposition/convolution algorithm.^(1)^ Sixteen parameters are available to adjust and are fitted to sets of PDD curves and profiles. Wedges and compensators have an additional modified scatter factor, MSF, where the fluence is multiplied by a factor 1+MSF×L, where L is the length of the primary ray through the modifier. Our commissioning of Pinnacle for the brass compensators consisted of adjusting only the two parameters, MSF and ρ. This has the advantage of maintaining the integrity of the open‐beam model. The same Pinnacle model can be used for all patient plans and it can be used to generate a plan using mixed fields of IMRT and 3D conformal. If a separate model were used exclusively for brass compensators, this would introduce a potential for error in the clinic. In cases where certain IMRT fields do not require a high level of modulation, the brass thickness required could be minimal or zero. In such cases, one would want the beam characteristics to collapse to the open‐beam model. In the case of .decimal compensators, there is a minimum base thickness of 0.6 cm.

Another difference in our methodology, compared with other published works, is our optimization of MSF and ρ based on the $\chi^{2}$ values of the brass attenuation curve versus those generated from Pinnacle, as shown in Fig. 1. This ensures that all brass thicknesses are considered in the optimization process. We have used a limited parameter search method varying MSF and ρ in turn to arrive at optimal values. A simultaneous 2D parameter search algorithm could yield further optimized results; however, based on the data presented, a dramatically different result is not expected. Whilst one would prefer an automated 2D search, such optimization algorithms are not available in the Pinnacle beam modeling system and, therefore, sequential manual searches bracketing physically relevant values of brass density were used.

Figures 2–6 show clear minima, indicating the robustness of our optimization method. In Fig. 5, there is a small spread in the minima as a function of depth in water, with the average at the minima $\rho =7.9\;g/{\text{cm}}^{3}$ for a depth of 10 cm, or D(10). Figure 6 shows a spread in minima from $7.3–8.1\;g/{\text{cm}}^{3}$ for field sizes of $5\times 5\;{\text{cm}}^{2}$ and $20\times 20\;{\text{cm}}^{2}$. The average ρ value among the curves is $7.80\;g/{\text{cm}}^{3}$. This is very close to $\rho =7.83\;g/{\text{cm}}^{3}$, the average ρ value among the curves in both Figs. 5 and 6, where all field sizes and depths are included.

Figures 5 and 6 show a trend where the smaller field sizes result in Pinnacle requiring a larger optimization parameter of density, ρ. This is likely due to the smaller field sizes having a higher percentage of their photons passing through the central part of the flattening filter, resulting in a harder beam incident on the brass slab.

Pinnacle's model parameters were also optimized using results from our MapCHECK device. Figures 7 and 8 show optimization data that give minima for $\rho =7.8\;g/{\text{cm}}^{3}$ and $\text{MSF}=0.1\;{\text{cm}}^{-1}$. These values are very close to the optimum values obtained from the brass slab data $\left(\rho =7.83\;g/{\text{cm}}^{3},\text{MSF}=0.001{\text{cm}}^{-1}\right)$. The slight variation is likely due to the increased scatter from compensators that have more "hills and valleys" in their structure than would a flat slab of brass. These values compare well with literature values from Opp et al.,^(4)^ who obtained $\rho =8.25\;g/{\text{cm}}^{3}$ and $\text{MSF}=0.11\;{\text{cm}}^{-1}$, albeit those data are for 6 MV. We were unable to find such data in the literature for 10 MV.

Brass compensators offer some advantages for IMRT plans, depending on the plan type and hardware available. Looking at a 95‐patient cohort from their record and verify system, Chang et al.^(5)^ have shown that, in general, the treatment times for compensators are quicker than segmental MLC delivery. An MU comparison by Buckey et al.^(10)^ show that MUs for brass are on average 20 MU less than MLC. Javedan et al.^(11)^ have studied the buildup dose for 6 MV, concluding that compensators give 7% less buildup dose than MLC IMRT plans due to beam hardening within the brass, resulting in fewer low‐energy photons in the treatment beam. For a moving target, Ehler et al.^(12)^ studied the temporal uniformity of the delivered dose and found that solid compensators perform better than MLC in these situations. For clinics without MLC available, brass compensators offer the ability to implement an IMRT program with very reliable planning and dose distribution.

## V. CONCLUSIONS

We present here a rigorous method to commission the Pinnacle treatment planning system for brass compensators, with examples shown for brass compensators from .decimal Inc. Our method is unique in two ways: we optimize only the two adjustable parameters that are not used in the open‐beam model, thus preserving the open‐beam model characteristics for fields not requiring intensity modulation, and/or for spatial locations within an IMRT field that the TPS requires zero attenuation. Secondly, our method optimizes the adjustable parameters based on the attenuation curve of a series of brass slabs using a $\chi^{2}$ analysis.

Our method shows that the Pinnacle treatment planning system parameters are optimized with values for $\text{MSF}=0.1\;{\text{cm}}^{-1}$, and density $\rho =7.8\;g/{\text{cm}}^{3}$ when using brass compensators for a Varian 10 MV beam. These values agree well with the work by Opp et al.,^(4)^ who report an $\text{MSF}=0.11\;{\text{cm}}^{-1}$, and a $\rho =8.25\;g/{\text{cm}}^{3}$ for a Varian 6 MV beam.

## Supporting information

Supplementary Material

Supplementary Material

Supplementary Material

Supplementary Material
