# Supplementary figures and images for: Quantitative analysis of brass compensators for commissioning of the Pinnacle planning system for IMRT
Source: J Appl Clin Med Phys. 2015 Nov 8;16(6):130–8. doi: 10.1120/jacmp.v16i6.5531 (PMC5690995; doi:10.1120/jacmp.v16i6.5531)

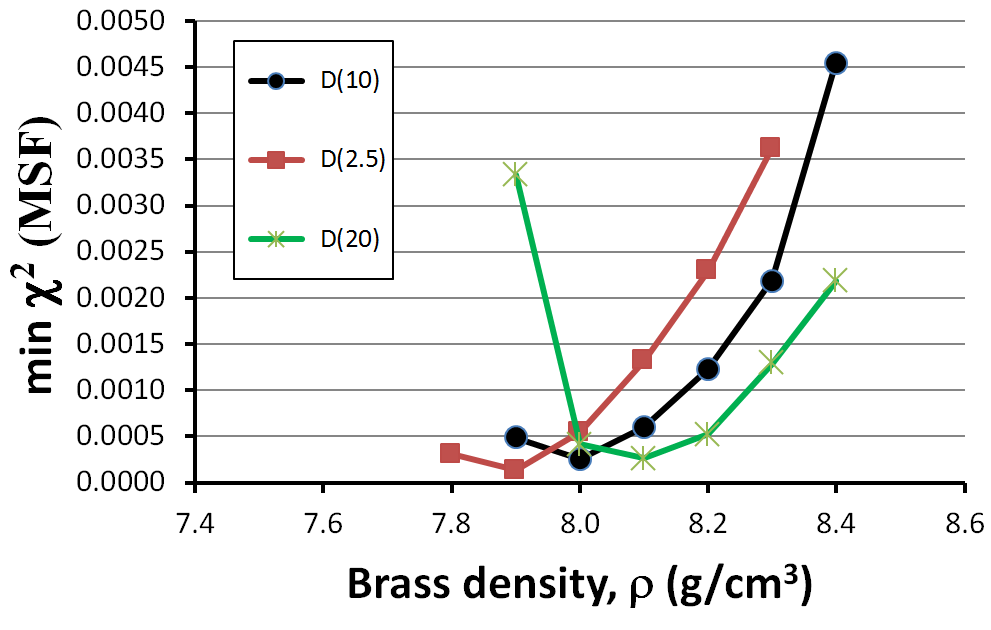

Supplement: Supplementary file 1 — Supplementary Material [file ACM2-16-130-s001.png]

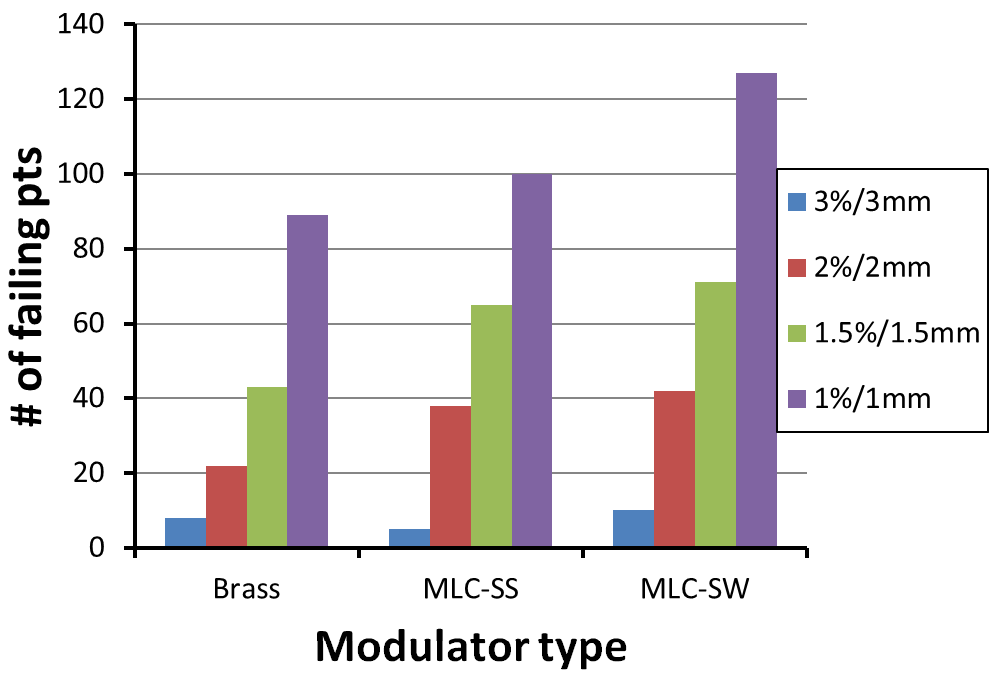

Supplement: Supplementary file 2 — Supplementary Material [file ACM2-16-130-s002.png]

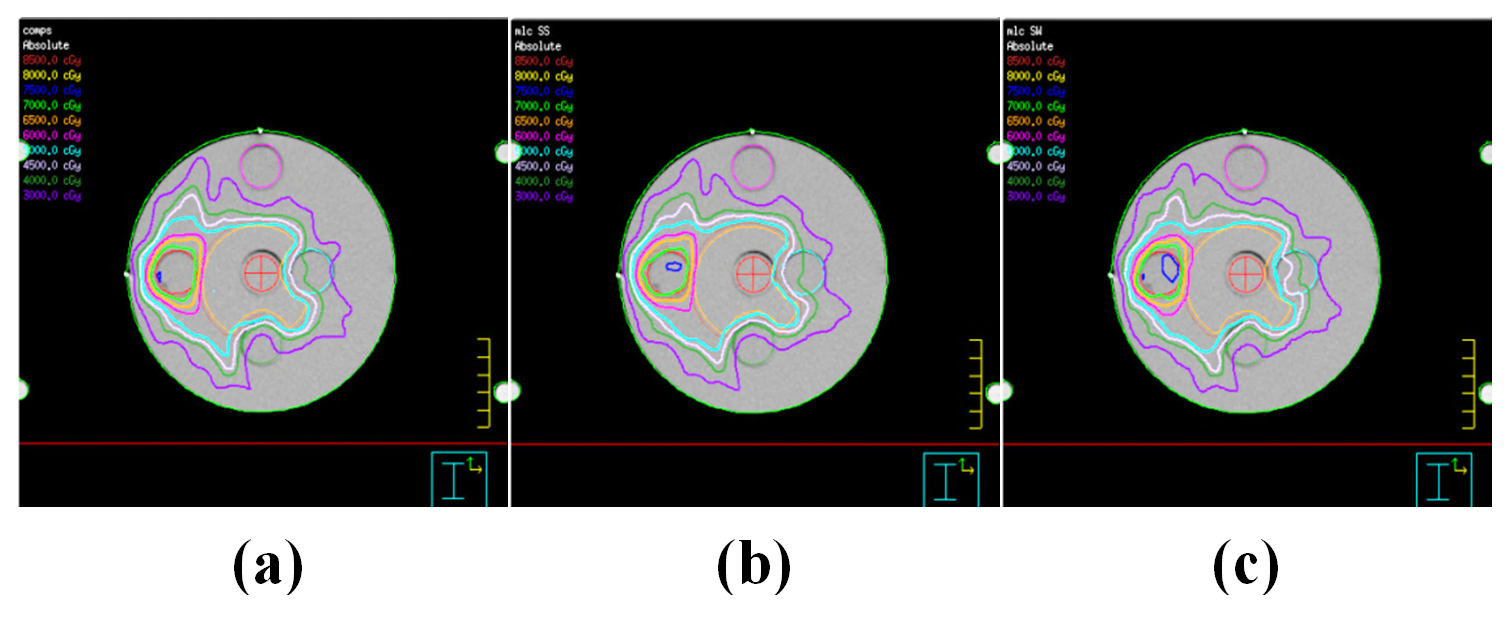

Supplement: Supplementary file 3 — Supplementary Material [file ACM2-16-130-s003.png]

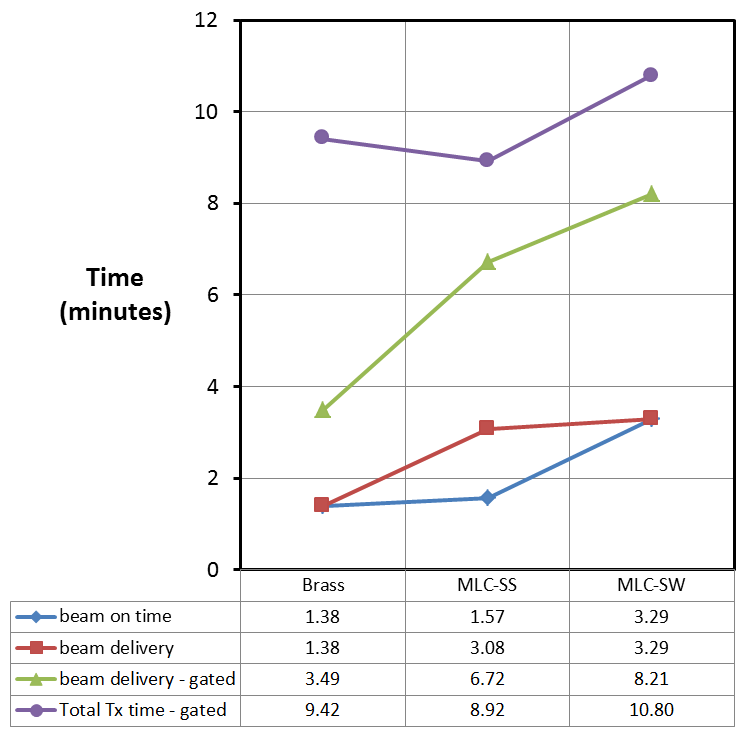

Supplement: Supplementary file 4 — Supplementary Material [file ACM2-16-130-s004.png]
